# Torsion of Pulmonary Sequestration

**DOI:** 10.1016/j.atssr.2025.04.005

**Published:** 2025-05-05

**Authors:** Yoshifumi Hirata, Kohei Hashimoto, Takahiro Tsuruta, Kazuharu Suda, Haruhiko Kondo

**Affiliations:** 1 Division of Thoracic Surgery, Kyorin University, Tokyo, Japan

**Figure 1:**
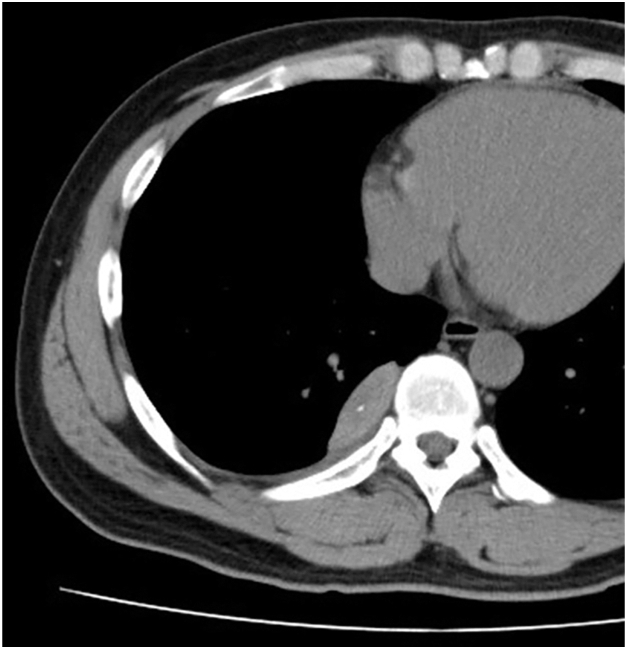

**Figure 2:**
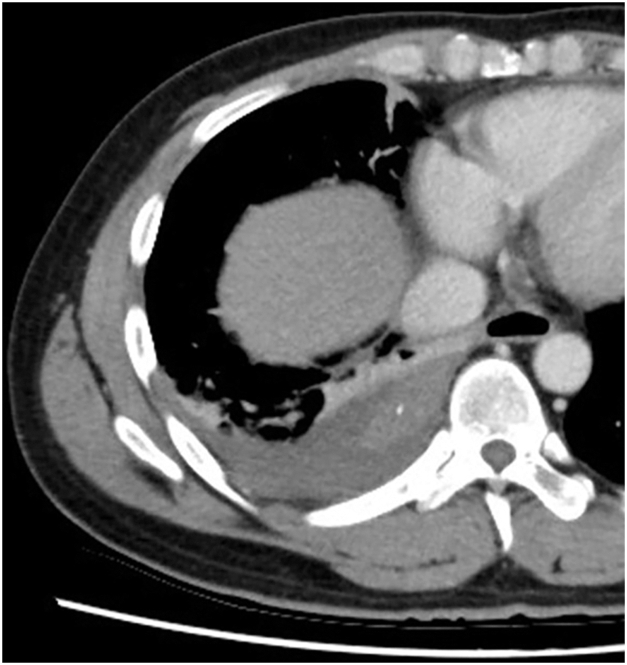

**Figure 3:**
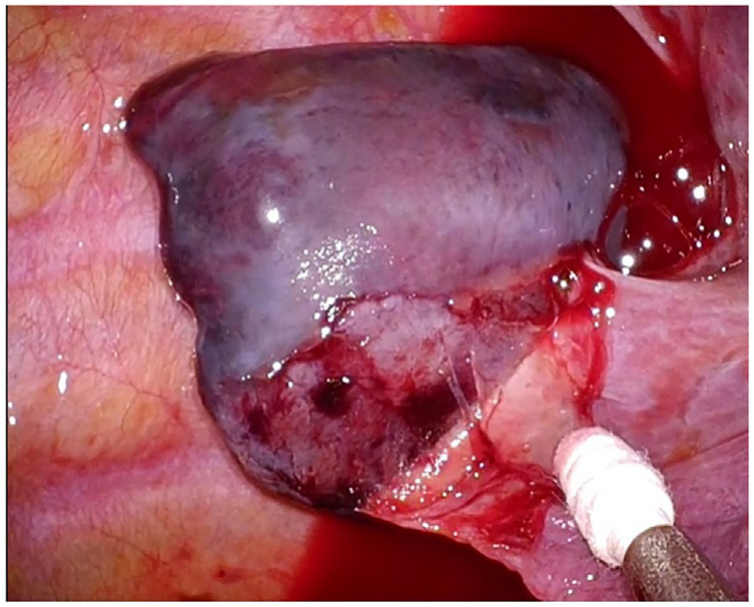

A 37-year-old man was admitted with sudden onset of back pain. Chest computed tomography revealed a spindle-shaped nodule adjacent to the right paravertebral body and pleural effusion (Figure 1). During workup, the pleural effusion increased, and the lesion was found to be pedunculated and floating in the pleural effusion (Figure 2), not typical of a mediastinal tumor. Although no aberrant vessels were observed, we suspected torsion of an extralobar pulmonary sequestration (EPS) and decided to perform surgery. During surgery, we found hemorrhagic pleural effusion and resected the mass (Figure 3), finding no obvious blood vessels, although a mediastinal attachment was carefully clipped (Video). There were no complications. On pathologic examination, it was a hemorrhagic necrosis of EPS.

EPS is typically found in children with other congenital malformation, and adult cases are rare.^1^ There are few reports of resected case of EPS for suspected torsion.^2^ When torsion occurs, it is difficult to identify the anomalous vessels that characterize EPS. One should be aware of the possibility of aberrant vessels during surgery in similar cases.
